# Controversies in the Management of Endometrial Cancer

**DOI:** 10.1155/2010/894587

**Published:** 2010-08-19

**Authors:** Enrique Hernandez, Andreas Obermair, Paul J. Hoskins, Javier F. Magrina, Robert Mclellan

**Affiliations:** ^1^Division of Gynecologic Oncology, Department of Obstetrics, Gynecology and Reproductive Sciences, Pathology and Laboratory Medicine, Temple University School of Medicine, Philadelphia, PA 19140, USA; ^2^Queensland Centre for Gynaecological Cancer, University of Queensland, QLD4029, Australia; ^3^Division of Medical Oncology, BC Cancer Agency, Vancouver Cancer Center, BC, Canada V5Z 4E6; ^4^Department of Obstetrics and Gynecology, Mayo Clinic Arizona, AZ 85054, USA; ^5^Department of Gynecology, Lahey Clinic, MA 01805, USA

Endometrial cancer is the most common gynecologic malignancy among women in developed countries. Most are diagnosed at stage I and their probability of surviving the disease is excellent. Most women at diagnosis are older, obese, and have multiple comorbidities. These factors need to be considered when treating patients with this highly curable disease. When recurrences occur at the vaginal apex, almost 50% are salvaged with radiation therapy with or without surgical excision. However, treatment of nodal or distant recurrences or of advanced disease at diagnosis is more challenging. 

One of the challenges faced by those treating this disease is how to identify those women who are at risk of having occult metastatic disease at initial diagnosis and to provide effective adjuvant therapy that would prevent recurrences in this select group of patients while minimizing therapy-induced morbidity.

In this special issue on endometrial cancer, the authors tackle some of these issues. The articles accurately and succinctly summarize the current approach to the treatment of this disease and looks at the future by discussing possible novel interventions for the treatment of advanced or recurrent disease. The controversies in the surgical management of endometrial cancer are discussed, as are the minimally invasive surgical techniques.

The international group of authors and editors provide a worldwide perspective about areas of agreement, topics of controversies, and issues that need yet to be clearly defined. Authors from The Netherlands discuss the controversies in evaluating women with postmenopausal bleeding. They weight the convenience, but possible decreased accuracy, of transvaginal ultrasound against the invasiveness of endometrial biopsy, hysteroscopy, and D&C. Another group from The Netherlands, using their national pathology database, demonstrates the prevalence of synchronous ovarian and endometrial adenocarcinomas.Authors from Indonesia thoroughly discuss the genetics of endometrial cancer, while authors from Bologna, Italy address controversies in the surgical treatment of endometrial carcinoma to include the laparoscopic and robotic-assisted approaches. The group from the University of Toyama, Japan presents a retrospective analysis of 83 women with endometrial carcinoma who by pre- and intraoperative assessment was considered to be at low risk for recurrence. These women underwent a total abdominal hysterectomy and bilateral salpingo-oophorectomy without lymphadenectomy. The authors report a 98% 5-year survival. The authors from China present a succinct but comprehensive review of endometrial carcinoma, as do the group from the Catholic University of Sacred Heart in Rome and Temple University in Philadelphia. The authors from the University of Texas in San Antonio discuss the differences in the molecular profile of type I and type II endometrial carcinoma, as well as the molecular changes observed in endometrial sarcomas. The authors from Oita University, Japan; Utrecht, The Netherlands; and the University of Florence, Italy discuss possible novel approaches to the treatment of advanced or recurrent endometrial adenocarcinoma. And finally, authors from Greece and the United Kingdom summarize the evidence for adjuvant and molecularly targeted therapy of endometrial cancer.

In this special issue of *Obstetrics and Gynecology International*, the reader will conveniently find a comprehensive summary of the state-of-the-art diagnostic strategies, biology, and evidence-based treatment of endometrial cancer from an international perspective.

*Enrique Hernandez* *Enrique Hernandez* *Andreas Obermair* *Andreas Obermair* *Paul J. Hoskins* *Paul J. Hoskins* *Javier F. Magrina* *Javier F. Magrina* *Robert Mclellan* *Robert Mclellan*

